# Lost or fond? Effects of nostalgia on sad mood recovery vary by attachment insecurity

**DOI:** 10.3389/fpsyg.2015.00773

**Published:** 2015-06-10

**Authors:** Sarah R. Cavanagh, Ryan J. Glode, Philipp C. Opitz

**Affiliations:** ^1^Laboratory for Cognitive and Affective Science, Department of Psychology, Assumption CollegeWorcester, MA, USA; ^2^Emotion and Cognition Laboratory, Davis School of Gerontology, University of Southern CaliforniaLos Angeles, CA, USA

**Keywords:** nostalgia, attachment, emotion, emotion regulation, sadness

## Abstract

Nostalgia involves a fond recollection of people and events lost to time. Growing evidence indicates that nostalgia may ameliorate negative affective states such as loneliness and boredom. However, the effect of nostalgia on sadness is unknown, and there is little research on how social connectedness might impact nostalgia's effects. Grounded in a theoretical framework whereby people with lower levels of attachment insecurity benefit more from nostalgia, we exposed participants to a mortality-related sad mood and then randomly assigned them to reflect on a nostalgic or an ordinary event memory. We examined changes in mood and electrodermal activity (EDA) and found that nostalgic versus ordinary event memories led to a blunted recovery from sad mood, but that this effect was moderated by degree of attachment insecurity, such that participants with low insecurity benefited from nostalgia whereas people with high insecurity did not. These findings suggest that nostalgia's benefits may be tied to the degree of confidence one has in one's social relationships.

## Introduction

“True joy is a profound remembering; and true grief the same.”—Clive Barker

Nostalgia is an intriguing phenomenon. On the one hand, nostalgia can be positive, imbued with a rosy glow of familiarity and belongingness. On the other hand, it can be negative, accompanied by longing, loss, and frustrated desire. Nostalgia often melds both positive and negative experiences. For instance, one of our participants shares his nostalgic reflection^1^:

“As a family we went to Moosehead Lake after my brother had passed away. My family saw this vacation as a way to escape the feelings of tragedy. It was warm and sunny and we were able to play in our bathing suits. For an hour things felt normal and we were happy. I will always remember that day as the last day I spent with my family before it truly fell apart.”

Fitting its blended nature, this nostalgic reflection involves both positive (unity, fun) and negative (death, tragedy) elements. In an attempt to elucidate the prototypical nature of nostalgic experiences, Hepper et al. (2012) conducted seven experiments suggesting that nostalgia is a complex blend of both cognition and affect, characterized by recalling one's past experiences with central prototypical features of personal meaning, feelings of fondness, and a “rosy glow.”

Nostalgia is rather common. A study by Wildschut et al. (2006) revealed that 79% of their undergraduate sample endorsed feeling nostalgic “once a week or more.” Negative affect, particularly loneliness, was the most reported elicitor of nostalgia. The authors also demonstrated that a negative mood induction resulted in higher state nostalgia than a positive or neutral induction. Subsequent research has supported the idea that a variety of negative affective states trigger nostalgic recollections, including social exclusion (Seehusen et al., 2013), boredom (van Tilburg et al., 2013), and meaninglessness (Routledge et al., 2011).

In terms of the *content* of nostalgic reflections, Wildschut et al. (2006) found that these reflections contain more positive than negative elements and that they feature the self embedded in a close social context. A “redemption” sequence (negative, then positive) is far more common than a “contamination” sequence (positive, then negative). Moreover, among participants asked to list desirable and undesirable features of nostalgia, 33% offered positive affect as a desirable feature, whereas 40% offered sadness as an undesirable feature.

Nostalgia therefore appears to involve an affective sequence whereby negative emotions trigger a nostalgic reverie, which is most often positive, and which ends on a redemptive (albeit often bittersweet) note. This sequence suggests that nostalgia may be instrumental in modulating negative affective states like sadness, either voluntarily or automatically. Indeed, Stephan et al. (in press) propose a theoretical model and supporting evidence suggesting that nostalgic experiences may serve to regulate affect. This model proposes that aversive experiences activate nostalgia, which then amplifies positive affective states, thus reflecting “a broader capacity of nostalgia to offset psychological distress and maintain psychological equanimity” (p. 5). In proposing such, the authors suggest that the negative affect automatically triggers the nostalgic memory. There is a rich literature supporting the idea that many of the processes we engage in to regulate our emotions occur involuntarily and even without conscious awareness (Mauss et al., 2007), and evidence that negative affective states in particular seem to elicit a tuning in toward positive information in undergraduate participants (DeWall et al., 2011).

Importantly, these associations between nostalgia and recovery from negative affective states may be impacted by the broader social context of the nostalgic reverie. For example, in the reflection shared by our participant above, it is evident that this participant felt that at least some of the social connections present in the nostalgic memory had since been irrevocably lost. It may be that he felt worse following his reflection than would someone who trusts that the social connections associated with his/her nostalgic reflection will extend into the future. Thus, if perceptions of social connectedness are a central feature of nostalgia, the degree of trust people can place in their social networks may influence the effect nostalgia has on affective states.

Nostalgia has indeed been found to increase feelings of social connectedness (Hepper et al., 2012). Although social others are of course not suddenly present during nostalgic recall, they do become present in mind. Aptly, Hertz (1990) termed this “peopling one's mind.” Supporting this peopling of the mind during nostalgia, Zhou et al. (2008) observed an indirect effect whereby loneliness increased perceived social support by way of increasing nostalgia. In addition to enriching perceptions of social connectedness, Routledge and colleagues provide evidence that nostalgia (both state and trait) may blunt the effects of terror primed by perceptions of mortality, and that it does so through enhancing a sense of meaning in life (Routledge et al., 2008; Juhl et al., 2010). Finally, van Tilburg et al. (2013) provided evidence that nostalgia also enhances a sense of meaning in the face of induced and dispositional boredom.

These data all suggest that nostalgia can be adaptive, diminishing unpleasant feelings associated with one's own death, and enhancing a sense of meaning. However, an extensive literature suggests that there are large individual differences in whether and to what extent people regulate their affective states, and also whether these attempts are successful (John and Eng, 2014). Considering this, alongside our reasoning that people's broader perception of their social context may inform the emotional outcomes of nostalgia, we propose a theoretical model in which the security of one's attachments may predict the effects of nostalgia on mood.

Adult attachment conceptualizes degree of trust in social relationships (Mikulincer et al., 2003), which grew out of Mary Ainsworth's classic literature (Ainsworth et al., 1978) on attachment between children and their caregivers. People with more secure attachments may benefit from nostalgia more than people with insecure attachments. If you distrust the reliability of your social relationships, calling them to mind may have little beneficial effect. Indeed, Wildschut et al. (2010) illustrated that the relationship between loneliness and nostalgia (including its reparative effects) may be particular to people low in insecure attachment.

Although work on nostalgia and its emotional implications has burgeoned in recent years, there are still notable gaps in our understanding of this elusive state of being. First, nearly all of the work investigating the nature of nostalgia has relied solely on self-reported measures of affect. While the work is commendable, thorough, and theoretically meaningful, studies including more objective measures (e.g., physiological response) are needed, as self-report is an important variable but one that is limited by degree of insight and numerous reporting biases.

Second, we do not know if nostalgia may serve to reduce negative affect other than mortality terror and boredom. To our knowledge, there has been no experimental investigation of whether nostalgia affects sadness, even though sadness is one of the most common negative emotions (Carstensen et al., 2000), and is highly relevant to depression, a form of mental distress with devastating costs to the individual and to society (Murray and Lopez, 1996).

These gaps stand in the way of a full understanding of nostalgia's role in people's emotional lives. Clarifying these open questions could refine our theoretical models of nostalgia and potentially pave the way for future applications in therapeutic contexts. We conducted the present study to test whether calling to mind a nostalgic versus an ordinary event memory would lead to greater recovery from a sadness induction and to evaluate whether attachment insecurity moderated these effects. We focused specifically on mortality-related sadness given the past literature on nostalgia and mortality terror and because the theme of threatened social ties (i.e., loss through death) might be a context in which attachment insecurity might be particularly relevant.

Situated in our review of the past literature and our theoretical model whereby the degree to which one trusts social attachments should impact the effect of nostalgic reflection on a sad mood state, we predicted that: (1) overall, following a mortality-related sad mood induction, people would feel better (i.e., higher decreases in sadness and increases in happiness) when this induction was followed by a nostalgic versus an ordinary event reflection, but that (2) this effect would be moderated by insecurity of attachment, such that people higher in attachment insecurity would not exhibit this nostalgia-related benefit.

## Materials and methods

### Participants

Seventy-one Assumption College undergraduates participated in this study (43 female; 51 Caucasian, age *M* = 20.19, *SD* = 2.00). The second- and third-largest ethnic groups self-reported as American Indian/Alaskan (six participants) and Hispanic/Latino (five participants). A power analysis for a linear regression analysis with a medium *a priori* effect size of f^2^ = 0.15, power (1-beta) of 0.80, two predictors, and alpha = 0.05 suggested a necessary N of 68 to detect effects. We are therefore well-positioned to detect effects of interest in our hypothesis tests below. Participants were randomized to one of two memory conditions (nostalgia, ordinary event), described below. All procedures were approved by the Assumption College Institutional Review Board, and participants provided written informed consent.

### Memory reflection

For the nostalgic/ordinary event memory reflections, we followed the methods of Hepper et al. (2012). Participants randomly assigned to the nostalgia condition were given a list of the 12 prototypical features of nostalgia (reminiscence, keepsakes, dwelling, rose-tinted memories, familiar smells, wanting to return to the past, family/friends, longing, feeling happy, childhood, emotions, personal) and asked to “bring to mind an event in your life that is relevant to or characterized by at least five of these features.” Also as specified by Hepper and colleagues, participants randomly assigned to the ordinary event memory reflection were encouraged to “bring to mind an ordinary event in your daily life. Specifically, try to think of a *past* event that is ordinary”. Participants were asked to draw forth a memory appropriate to their condition and alert the experimenter when they were ready. In both conditions they were asked to spend 2 min quietly reflecting on the memory, following which they were asked to write a short narrative describing the event they chose. We submitted these narratives to analysis in LIWC (Linguistic Inquiry and Word Count; Francis and Pennebaker, 1993) software to obtain the following scores for these narratives: overall word count, and number of words related to: past, present, future, social words, positive emotion, negative emotion, sadness, and death. See Table 1 for descriptive statistics on these and all major study variables.

**Table 1:** **Descriptive statistics (means, standard deviations) for major study variables, separately by memory reflection condition**.

**Variable**	**Nostalgia mean (SD)**	**Ordinary event mean (SD)**
**SADNESS RATINGS**		
Pre-clip sad	0.343 (0.765)	0.200 (0.531)
Post-clip sad	2.800 (0.797)	2.686 (1.231)
Post-memory sad	1.629 (1.374)	0.600 (0.775)
**HAPPY RATINGS**		
Pre-clip happy	2.229 (1.031)	2.171 (1.043)
Post-clip happy	0.743 (0.886)	0.943 (0.938)
Post-memory happy	2.200 (1.410)	1.886 (1.022)
**EDA**		
Neutral aquatic	11.238 (9.356)	12.489 (10.824)
Sad film clip	12.836 (10.459)	14.320 (11.426)
**NARRATIVE CONTENT**		
Word count	67.690 (40.097)	52.600 (41.383)
Past words	5.116 (4.685)	3.599 (5.218)
Present words	1.191 (2.147)	2.610 (4.210)
Future words	0.865 (1.980)	0.238 (0.654)
Social words	4.490 (6.357)	2.045 (3.921)
Positive emotion words	3.180 (2.946)	2.161 (2.202)
Negative emotion words	1.259 (1.606)	0.621 (1.341)
Sad words	2.249 (2.779)	0.697 (1.929)
Death words	5.349 (5.370)	2.437 (4.565)
Attachment insecurity	30.324 (9.993)	31.426 (10.877)

### Mood ratings

Participants rated their current mood on 11 dimensions (relaxed, sad, amused, energetic, anxious, happy, tired, fearful, irritated, content, bored) on 5-point Likert style scale four times: at Baseline, Pre-Sad Clip, Post-Sad Clip, and Post-Memory. Given the focus of this paper on people's reactions to unpleasant stimuli specifically targeting loss we analyzed the sad and happy items separately rather than more global negative and positive affect ratings^2^. We did not collapse sad and happy into a bipolar mood scale due to our a-priori interest in investigating nostalgia's effects on both negative and positive affect separately, and because the negative correlations between these items were only weak to moderate (see Table 2).

**Table 2:** **Pearson correlations (and** ***p*****-values) between happy and sad ratings at designated times of measurement**.

	**r (p)**
Pre-clip	−0.188 (0.199)
Post-clip	−0.260 (0.030)
Post-memory reflection	−0.484 (<0.0001)

### Psychophysiology

Electrodermal activity (EDA) was selected as a pure measure of sympathetic activation of the autonomic nervous system. Two disposable Ag/AgCl electrodes pregelled with 0.5% chloride isotonic gel (1 cm circular contact area) were attached to the distal phalanges of the index and middle fingers on the non-dominant hand. EDA level was recorded with DC coupling and constant voltage electrode excitation at 35 Hz (sensitivity = 0.7nS). Offline, EDA was smoothed with a 1 Hz low-pass filter, decimated to 10 Hz, and further smoothed with a 1-s prior moving average filter. Offline data filtering and reduction were completed using using ANSLAB routines (Wilhelm and Peyk, 2005) routines in Matlab (Mathworks, Natick, MA).

We recorded EDA during a neutral aquatic clip (to serve as a neutral comparison to the sad clip) and during the sad film clip. We collected EDA for the entirety of the film clips, and used the mean skin conductance level as our dependent measure. Participants with average EDA levels greater than three standard deviations from the grand mean were excluded^3^.

In a review of the literature on the autonomic profiles of various emotional states, Kreibeig and colleagues (Kreibig, 2010) found that when sadness is loss-related and induces crying, sympathetic activation (including increases in skin conductance level) is typically observed, whereas for non-loss, non-crying sadness, sympathetic withdrawal (including decreases in skin conductance level) is typically observed. Studies using film clips were represented in both of these categories, though more studies using film clips found deactivation than activation (for a representative exception, see a paper by the same author (Kreibig et al., 2007) using loss-related film clips). Given the strong theme of loss present in our film clip (parent losing a child) and the anecdotal information that several of our participants used tissues at the conclusion of the sad film clip, we predicted a greater elevation of electrodermal response in the sad film clip measurement than the neutral baseline.

### Adult attachment

Adult attachment was assessed with 13 items from the Relationship Scale Questionnaire (RSQ; Griffin and Bartholomew, 1994; see Supplementary Material for included items). Brennan et al. (1998) suggest that the best fitting model for adult attachment involves two dimensions: *anxiety*, involving fear of separation and an excessive need for approval, and *avoidance*, involving fear of intimacy and an excessive self-reliance. People scoring high on either (or both) dimension(s) are considered to have insecure attachment, whereas people scoring low on both have secure attachments (Wei et al., 2007). We included nine items relevant to anxious attachment (e.g., “I worry about being abandoned”) and four items relevant to avoidant attachment (e.g., “I worry about others getting too close to me”). Items were rated on a 5-point scale, from “not at all like me” to “very like me.^4^”

### Procedure

Following consent and psychophysiological setup, all participants viewed a 5 min, 44 s neutral aquatic clip to establish a low-arousal baseline. All participants then rated their mood (Pre-Sad Clip) and viewed a 6 min, 07 s film clip taken from the movie *My Sister's Keeper*. This clip portrays a mother's last conversation with her dying teenage daughter, and concludes with the girl's death. Participants rated their mood again (Post-Sad Clip), and then were randomly assigned to engage in either a nostalgic or an ordinary event reflection. Following the memory reflection, participants rated their mood a final time (Post-Memory) and then filled out questionnaires assessing demographic information and the adult attachment measure via SurveyMonkey.com.

## Results

### Preliminary analyses

#### Did we induce a sad mood state?

To determine whether we successfully induced a sad mood state with the film clip, we submitted participants' mood ratings Pre-Sad Clip and Post-Sad Clip to paired samples *t*-tests. This analysis revealed significant increases in sadness from pre- to post- the sad clip, *t*_(69)_ = 18.582, *p* < 0.0001, *d* = 2.858, and reductions in happiness, *t*_(69)_ = 10.959, *p* < 0.0001, *d* = 1.310. Paired samples *t*-tests also revealed that average skin conductance levels were significantly higher during the sad clip than during the neutral baseline clip, *t*_(61)_ = 5.581, *p* < 0.0001, *d* = 0.162. Moreover, there was a trend toward a positive association between skin conductance level during the sad film clip and changes in rated sadness from Pre-Sad Clip to Post-Sad Clip, *r*_(62)_ = 0.227, *p* = 0.071. The direction of this association is consistent with the notion that sympathetic activation during the film clip is associated with increases in sadness participants experienced. Together, these findings suggest a successful induction of a sad mood state.

#### Did the memory reflection conditions differ from one another in content of the reflections?

To explore how the conditions differed in the narrative content of the reflections, we conducted a series of univariate ANOVAs where the between-subjects variable was memory condition and the dependent variables were overall word count, words per sentence, and number of words related to past, present, future, sociality, positive emotion, negative emotion, sadness, and death. The narratives did not significantly differ from one another in overall word count or number of words that related to past, present, or future, suggesting that participants in the two conditions recalled memories from the past and described them with a similar level of complexity (at least as measured by number of words used to describe said event). The two conditions also did not differ in the number of positive words or negative words used. However, people in the nostalgia condition reported narratives that contained more words related to death (*p* = 0.029, η^2^ partial = 0.081), sadness (*p* = 0.015, η^2^partial = 0.099), and a trend toward more words related to sociality (*p* = 0.080, η^2^ partial = 0.053).

#### Did the abbreviated measure of adult attachment yield valid psychometric properties?

Since we were using an abbreviated measure of a validated scale, we took two approaches to demonstrate that our measure had acceptable psychometric properties. First we examined all of the items together in an exploratory factor analysis to see how the items converged, and then we examined the internal consistency of the subscales and the overall measure using Cronbach's alpha. Both approaches suggested that after dropping one item that did not load well onto the main factor (“I am comfortable without close emotional relationships”), the items loaded onto a single factor. The best fit for the factor analysis, according to the coefficient values and visual inspection of the scree plot, was a single-factor solution. Moreover, Cronbach's alpha was moderate for the subscales (0.864 for anxious and 0.668 for avoidant), whereas it was extremely strong for the scale as a whole (0.904). Therefore, we conceptualized this scale as representing insecure attachment in general, with low scores representing more secure attachment, and high scores representing insecure (fearful-avoidant) attachment.

### Hypothesis testing

#### Did engaging in nostalgic vs. ordinary event memory reflections result in differential changes in self-reported mood or electrodermal activity?

**Figure 1:**
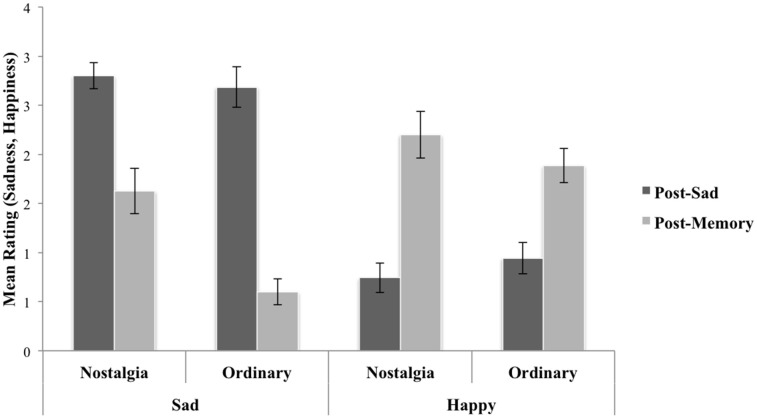
**Mean ratings of sadness and happiness following the sad film clip and following the memory reflection, separately by memory condition, illustrating that people in the nostalgic condition had smaller decreases in sadness from post-film clip to post-memory reflection than people in the ordinary event condition**. Participants also experienced greater elevations in happiness in the nostalgic condition, but this effect was only a trend (*p* = 0.072).

To test whether the nostalgic vs. ordinary event memory reflections resulted in differential changes in mood following the sadness induction, we submitted the mood ratings (sadness, happiness) to three 2 × 2 general linear models (GLMs) with one between-subjects factor (memory reflection: nostalgic, ordinary event) and one within-subjects factor (time: post-sad, post-memory). The GLM for sadness ratings revealed main effects of time, *F*_(1, 68)_ = 103.724, *p* < 0.0001, η^2^ partial = 0.604 (greater sadness after the film clip than after the memory reflection), a main effect of memory reflection, *F*_(1, 68)_ = 8.024, *p* = 0.006, η^2^ partial = 0.106 (collapsing across time, greater sadness in the nostalgia condition), and an interaction of time of assessment and memory reflection, *F*_(1, 68)_ = 8.173, *p* = 0.006, η^2^ partial = 0.107 (greater reductions in sadness in the ordinary event memory vs. the nostalgic condition, see Figure 1). The GLM for happy ratings revealed a main effect of time of assessment, *F*_(1, 68)_ = 72.479, *p* < 0.0001, η^2^ partial = 0.516 (greater happiness after the memory condition than after the film clip), no main effect of memory condition, *F*_(1, 68)_ = 0.069, *p* = 0.793, η^2^ partial = 0.001, and only a weak trend toward a significant interaction of time and memory condition, *F*_(1, 68)_ = 3.328, *p* = 0.072, η^2^ partial = 0.047 (higher increases in happiness in the nostalgia condition). The GLM for EDA revealed a main effect of time of assessment, *F*_(1, 59)_ = 37.068, *p* < 0.0001, η^2^ partial = 0.386 (greater EDA in the memory reflection than in the sad mood induction), no main effect of memory condition, *F*_(1, 59)_ = 0.055, *p* = 0.816, η^2^ partial = 0.001, and no interaction of time and memory condition, *F*_(1, 59)_ = 0.131, *p* = 0.718, η^2^ partial = 0.002.

#### Were individual differences in attachment insecurity predictive of mood change following memory reflection?

To test whether attachment insecurity moderated the relationship between memory condition and sadness recovery, we computed separate linear regression models using PROCESS in SPSS (Hayes, 2013) for sadness and happiness changes. For sadness, we used as the dependent measure difference scores computed as (Post-Film sadness ratings—Post-Memory Reflection sadness ratings), where higher scores indicate greater *reductions* in sadness (thus conceptualizing mood recovery). Conversely, for happiness, we used as the dependent measure difference scores computed as (Post-Memory sadness ratings—Post-Film sadness ratings), where higher scores indicate greater *elevations* in happiness (thus conceptualizing mood recovery).

The models were constructed such that memory condition predicted mood changes (changes in sadness, happiness). We entered relationship attachment as the moderator of this relationship to test whether the association between nostalgia condition and mood changes depends on the degree to which one trusts one's social relationships.

**Figure 2:**
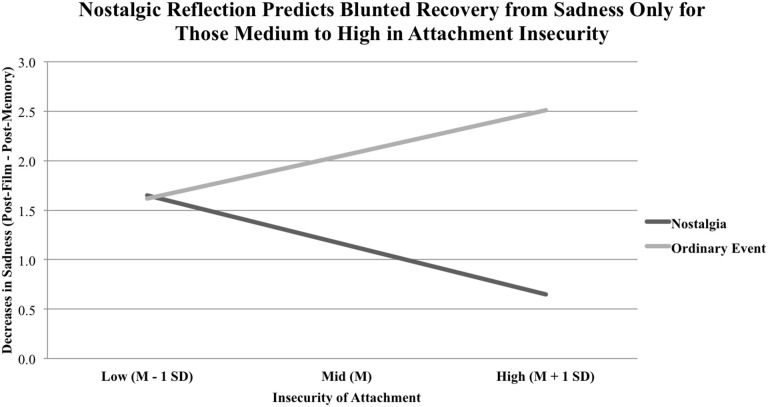
**Memory condition and attachment insecurity as predictors of decreases in sadness (Post-Film minus Post-Memory)**. Low, medium, and high attachment insecurity on the x-axis represent values that are one standard deviation below the mean, the mean, and one standard deviation above the mean, respectively. We observed an interaction of Attachment Insecurity × Memory Condition such that nostalgic condition (dark line) only predicted worse recovery from sadness for those with medium or high levels of attachment insecurity.

For sadness, the overall model was significant, *R*^2^ = 0.216, *F*_(3, 65)_ = 5.958, *p* = 0.001. Memory condition exhibited near-significant trend toward predicting lower sadness recovery, *b* = −1.9007, *p* = 0.056, 95% CI [−3.8529, 0.0516]. Moreover, levels of insecure attachment did predict lower sadness recovery, *b* = −0.1393, *p* = 0.006, 95% CI [−0.2372, −0.0415]. Critically, these two main effects were qualified by an interaction effect of Attachment Insecurity x Memory Condition, *b* = 0.0911, *p* = 0.004, 95% CI [0.0310, 0.1512], where nostalgic memory condition was associated with lower recovery from sadness for those medium to high in attachment insecurity, *b* = 0.9129, *p* = 0.004, 95% CI [0.2961, 1.5297] and *b* = 1.859, *p* = 0.0001, 95% CI [0.9791, 2.7397], respectively. Importantly, this was not apparent among participants low in attachment insecurity, *b* = −0.0336, *p* = 0.9392, 95% CI [−0.9085, 0.8414] (see Figure 2). This result supports our hypothesis that indulging in nostalgia results in lower recovery from mortality-related sadness *only* in the presence of higher levels of attachment insecurity.

**Figure 3:**
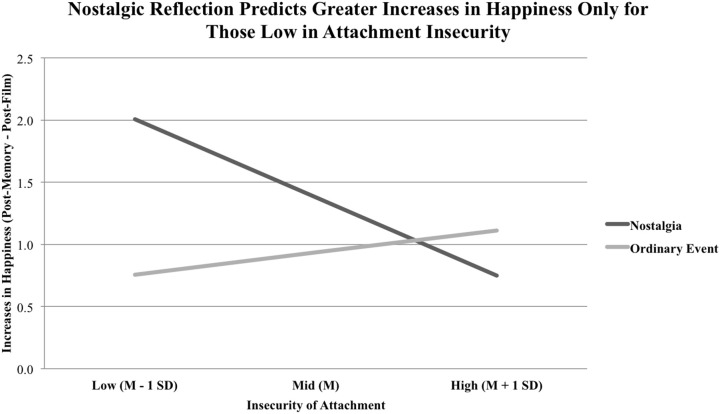
**Memory condition and attachment insecurity as predictors of increases in happiness (Post-Memory minus Post-Film)**. Low, medium, and high attachment insecurity on the x-axis represent values that are one standard deviation below the mean, the mean, and one standard deviation above the mean, respectively. We observed an interaction of Attachment Insecurity × Memory Condition such that nostalgic condition (dark line) predicted greater elevations of happiness only in those with low levels of attachment insecurity.

For happiness, the overall model was significant, *R*^2^ = 0.178, *F*_(3, 65)_ = 4.689, *p* = 0.005. Here, memory condition significantly predicted happiness elevation, *b* = −2.845, *p* = 0.001, 95% CI [−4.524, −1.167]. Moreover, levels of insecure attachment did predict lower happiness elevation, *b* = −0.138, *p* = 0.002, 95% CI [−0.222, −0.054]. Critically, these two main effects were qualified by an interaction effect of Attachment Insecurity × Memory Condition, *b* = 0.078, *p* = 0.004, 95% CI [0.026, 0.129], where memory condition was associated with higher elevations in happiness for participants low in attachment insecurity, *b* = −1.252, *p* = 0.002, 95% CI [−2.005, −0.500] but not for those medium to high in attachment insecurity, *b* = −0.444, *p* = 0.100, 95% CI [−0.975, 0.087] and *b* = 0.363, *p* = 0.958, 95% CI [−0.394, 1.120], respectively (see Figure 3). This result supports our hypothesis that indulging in nostalgia results in higher elevations of happiness following mortality-related sadness, but *only* for those low in attachment insecurity.

## Discussion

Engaging in a nostalgic reflection following a mortality-related sad mood resulted in significantly *lower* mood recovery (at least in terms of decreases in sadness) than engaging in an ordinary event memory. Considered by itself, this finding did not support our associated hypothesis. Importantly, however, this effect was moderated by insecurity of attachment, such that nostalgic reflections led to *worse* recovery in people with medium to high levels of attachment insecurity (lower decreases in sadness and no effect on happiness relative to the control condition) but led to *better* recovery in people with low levels of insecure attachment (higher elevations in happiness and no effect on sadness relative to the control condition).

### Nostalgic reflections led to lesser recovery from sad mood

Rather than nostalgic reflections benefiting participants' moods overall, we observed a relative blunting of recovery from sadness in the medium and high insecure attachment participants and no difference between nostalgic and ordinary event reflections in the low insecure attachment participants. There are several possible explanations for these effects. It may be that nostalgia is not an effective tool for regulating sadness. Its bittersweet nature may mean that while it can alleviate existential terror (Routledge et al., 2008) and boredom (van Tilburg et al., 2013), it is less effective for sadness, an emotion already associated with loss. Alternatively, it may be that the lingering of sadness combined with positive affect (we observed numerically higher rates of positive affect in the nostalgic condition, not lower, and people with secure attachments benefited from nostalgia in terms of happiness) is simply reflective of nostalgia's blended nature. Nostalgia may not nullify sadness, but rather introduce a poignant positivity to the still-present negativity.

Nostalgia might have differential effects on other, non-social-loss related negative emotions like anxiety or disgust. “Peopling one's mind” may only introduce some positivity to negative experiences when the negative experiences are specifically social. Subsequent examinations may benefit from a consideration of the effect of nostalgia on these non-social emotions.

Second, it may be that nostalgia *was* effective in regulating emotion (all participants experienced significant decreases in sadness following the memory reflection), but the ordinary event memory may have been *even more* effective. Distraction is a very effective form of emotion regulation (Sheppes and Meiran, 2007), and thinking of a relatively neutral past event from one's everyday life may have been a welcome distraction from sadness. While having a memory-based neutral control condition is important, future designs could implement additional controls to address distraction. Moreover, the nostalgic memory may have been more cognitively demanding than the ordinary event memory and thus been associated with slower recovery from sad mood. While the reflections did not differ in the number of “past” words used, given the nature of nostalgic memory, participants in the nostalgic condition were probably calling to mind memories of events deeper in the past than the ordinary event condition.

Third, it is unclear whether these observed relationships are unique to the experience of nostalgia. For instance, it could be that one would observe these relationships for any evocation of social experiences, not just ones characterized by nostalgia. Focusing on the “rosy glow” aspect of nostalgia, it could also be that these observed relationships would be present for any over-idealized mental simulation characterized by longing, such as fantasizing about an unrequited romance or imagining quitting one's responsibilities to live on a sailboat. Given our desire to clarify the effects of *nostalgic* experiences in particular, we did not test these other possibilities. Nonetheless, these considerations highlight the fact that nostalgia is a multifaceted experience, and we need more work to investigate which specific aspects (idealization, longing, memory, negativity/positivity, sociality) are contributing to the observed relationships, as well as work that distinguishes it from similar but distinct experiences such as regret (Gilovich and Medvec, 1995) or life longings (Scheibe et al., 2007).

### Effects of nostalgia on sad mood moderated by insecurity of attachment

When one considers the moderation effect, it appears that these main effects of nostalgia on mood recovery are being driven by those with medium to high levels of attachment insecurity. Underscoring Wildschut et al.'s (2010) past findings regarding loneliness, nostalgia's reparative effects may be reserved for those with the perception that their social connections are stable. Nostalgia may deliver warm, connected memories from the past in order to create feelings of safety and meaning for the future—but only if you can trust your relationships to remain stable into that unknown future. This finding joins a growing literature on retrospective and prospective mental simulation (Markman and Dyczewski, 2013) and is consistent with research by Cheung et al. (2013) suggesting that nostalgia might increase optimism for the future—but qualifies a boundary condition where this may only be true for those with secure attachments. In any consideration of therapeutic applications of nostalgia, especially for those struggling with losses such as bereavement, it might be worthwhile to first consider the security of a person's existing attachments so as not to inadvertently make people feel worse.

Of course, attachment insecurity is only one measure of the quality of one's social relationships. Size and quality of one's social network, perceived social support, and frequency of positive social interactions are just a few measures that could relate to either/both attachment insecurity and response to nostalgia. Moreover, past social experiences could relate to both current attachment insecurity and one's particular reaction to nostalgia. Any of these potential third variables could explain the observed relationship between attachment insecurity and nostalgia, or could contribute their own effects. Future research is indicated to tease out these complicated relationships between social history, social perceptions, and response to nostalgia.

### Strengths and limitations

Strengths of this paper include the experimentally controlled investigation of nostalgia's effects on a common and sometimes destructive mood (sadness), the inclusion of multiple channels of the emotional response (self-report, physiology), and the partial confirmation of our theoretical model predicting that nostalgia's ability to reduce negative affect and bolster positive affect after a sad mood induction may vary by one type of social connectedness (adult attachment).

In terms of limitations, we do not know whether our results are specific to sadness or generalizable to other forms of negative affect. Given that other researchers have found restorative effects of nostalgia on mortality terror (Routledge et al., 2008) and boredom (van Tilburg et al., 2013), these results may indeed be specific to mortality-related sadness. Future research will need to tease out which negative affective states are positively and negatively affected by nostalgia, including varieties of sadness not related to personal loss. Second, while we collected physiological data, our significant findings were all in the domain of self-report. As EDA is a relatively non-specific measure of autonomic arousal activity (Dawson et al., 2007), it may be that nostalgia's blend of both negative and positive emotion make this a non-ideal measure for assessing reaction to nostalgia. Third, our measure of attachment insecurity was limited in nature. Though our measure demonstrated high levels of internal consistency, these results need to be replicated with a full, psychometrically validated scale.

Fourth, we acknowledge that including our attachment measure after the sadness and memory reflections could have resulted in differential self-reported attachment levels by condition (i.e., state effects). However, we were concerned that had we included the attachment measure first, it may have primed participants to think about the security of their relationships *before* the sadness induction and memory reflection and thus altered their reactions to these manipulations. Moreover, in our design, participants engaged in a series of tasks between the memory reflection and completion of questionnaires, including writing out the content of the reflections, being detached from psychophysiological equipment, and the completion of mood ratings and other measures. Therefore, we had good reason to expect that these intermediate tasks would weaken or nullify any potential priming effects. Thankfully, the concern that our memory manipulations would differentially impact ratings of attachment insecurity did not manifest in the present data, as there was no suggestion of differences in attachment insecurity between the nostalgic and the ordinary event conditions, *F*_(1, 67)_ = 0.192, *p* = 0.663, η^2^ partial = 0.003. However, in future work we recommend that participants complete such measures in a pre-screening to avoid confounds in either direction.

Finally, while laboratory investigations of affective phenomena like nostalgia constitute an important first step, a true understanding of the role nostalgia plays in people's emotional lives will require more sophisticated methodology that explores the causes, correlates, and effects of nostalgia in an intra-individual design sensitive to the effects of situational context.

### Conclusions and future directions

In conclusion, the poignant nature of nostalgia and the likelihood that sadness may elicit memories of loss may mean that engaging in nostalgia leads to a lingering of sadness. Intriguingly, this effect seemed to vary by one's trust in relationships, such that those with insecure attachments responded more negatively to nostalgia and those with more secure attachments respond more positively. Future research should clarify whether these effects are specific to sadness, whether subtypes of insecure attachment relate differently to nostalgia's effects, and whether choosing a nostalgic memory based on the theme of the mood induction impacts the mood effects of the nostalgic reverie.

## Author contributions

SC, RG, PO designed, conducted, and analyzed the experiment. RJ took primary responsibility for data collection. SC took primary responsibility for the written work, with contributions from RJ and PO at multiple stages of manuscript preparation.

### Conflict of interest statement

The authors declare that the research was conducted in the absence of any commercial or financial relationships that could be construed as a potential conflict of interest.

## Footnotes

^1^Minor details changed to preserve anonymity. See Supplementary Material for additional representative reflections.

^2^Repeating our analyses using positive and negative affect revealed similar (albeit weaker in the case of negative) results as using the more targeted items. Moreover, while a multiple-item measure of sadness and happiness may have maximized our ability to detect important relationships, single-item measures of mood responses have been demonstrated as reliable and valid by previous research (e.g., Hürny et al., 1996; Zimmerman et al., 2006).

^3^We also collected corrugator electromyography, but due to multiple problems with the equipment, these data were not analyzed and are not presented.

^4^Other self-report measures were collected for a student thesis but as they are not relevant to the current paper, are not reported.
