# Supplementary material for: Lost or fond? Effects of nostalgia on sad mood recovery vary by attachment insecurity
Source: Front Psychol. 2015 Jun 10;6:773. doi: 10.3389/fpsyg.2015.00773 (PMC4461810; doi:10.3389/fpsyg.2015.00773)
Supplement: Supplementary file 1 [file SupplementaryMaterial.docx]

Appendix A

Additional Nostalgic Memory Reflections

The event was winning the league in soccer. I remember it as satisfying and the most fun I have ever had playing a sport. It was a night game on our home field in front of many fans. It was glorious, but it also marked the end of me playing soccer.

The event was when I left my house to go to move in to college. I was saying goodbye to my grandfather. He lives with us and recently had two strokes in June 2011. I always remember him being strong, and the past two months were very difficult to see him struggling. It took a lot to hold back tears, but I didn't want to cry in front of him or my mom. I kept thinking it might be the last time I saw him, and I still think that each time I go home and see him.

For Christmas and New Years my direct family and two other families rented a house in Vermont. Every night we would sled down the giant hill in the back yard, or snow board. We then would go inside for home cooked meals, Christmas desserts like apple pie and hot chocolate and just have fun family time. That year we all made a New Years Resolution to do things like this all the time and we have ever since. Smells like apple pie, cinnamon, hot chocolate, or pine bring me back to that winter and other fun family events after this.

Appendix B

Items Measuring Insecure Attachment

1. I find it difficult to depend on others.
2. I worry that I will be hurt if I allow myself to become too close to others.
3. I am not sure that I can always depend on others to be there when I

need them.

1. I worry about being alone.
2. I often worry that romantic partners don’t really love me and won’t want

to stay with me.

1. I find it difficult to trust others completely.
2. I worry about others getting too close to me.
3. I worry that others don’t value me as much as I value them.
4. People are never there when you need them.
5. My desire to merge completely sometimes scares people away.
6. I am nervous when anyone gets too close to me.
7. I worry about being abandoned.
